# Supplementary material for: Electrochemical CO2 reduction to high-concentration pure formic acid solutions in an all-solid-state reactor
Source: Nat Commun. 2020 Jul 20;11:3633. doi: 10.1038/s41467-020-17403-1 (PMC7371694; doi:10.1038/s41467-020-17403-1)
Supplement: Supplementary file 1 — Supplementary Information [file 41467_2020_17403_MOESM1_ESM.pdf]

Electrochemical CO_2 reduction to high-concentration pure formic acid solutions in an all-solid-state reactor

Lei Fan[1,2]†, Chuan Xia[1,3]†, Peng Zhu[1], Yingying Lu[2]*, Haotian Wang[1,4]*

[1]Department of Chemical and Biomolecular Engineering, Rice University, Houston, TX 77005, USA.
[2]State Key Laboratory of Chemical Engineering, Institute of Pharmaceutical Engineering, College of Chemical and Biological Engineering, Zhejiang University, Hangzhou 310027, China.
[3]Smalley-Curl Institute, Rice University, Houston, TX 77005, USA.
[4]Azrieli Global Scholar, Canadian Institute for Advanced Research (CIFAR), Toronto, 22 Ontario M5G 1M1, Canada.
†These authors contributed equally.
*Corresponding author Email: yingyinglu@zju.edu.cn (Y.L.); htwang@rice.edu (H.W.).

Supplementary Figures

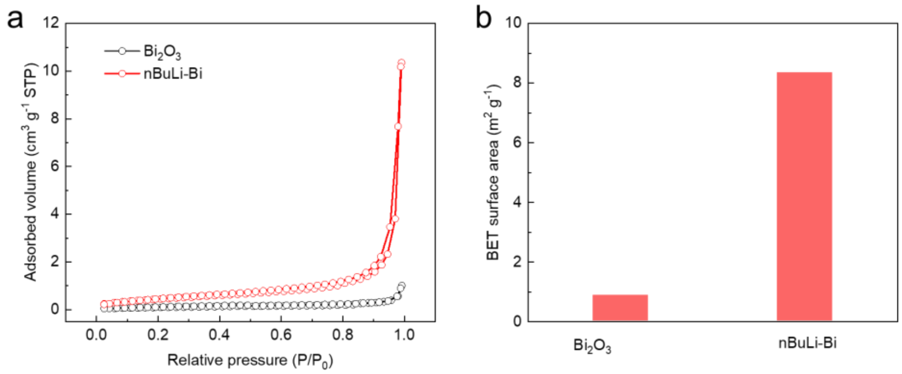

Supplementary Figure 1. BET characterizations of Bi_2O_3 and nBuLi-Bi. a, N_2 adsorption and desorption isotherms of Bi_2O_3 and nBuLi-Bi. **b**, BET surface areas of Bi_2O_3 and nBuLi-Bi. The BET surface area of nBuLi-Bi was much higher than pristine Bi_2O_3, which can provide more active sites for CO_2RR.

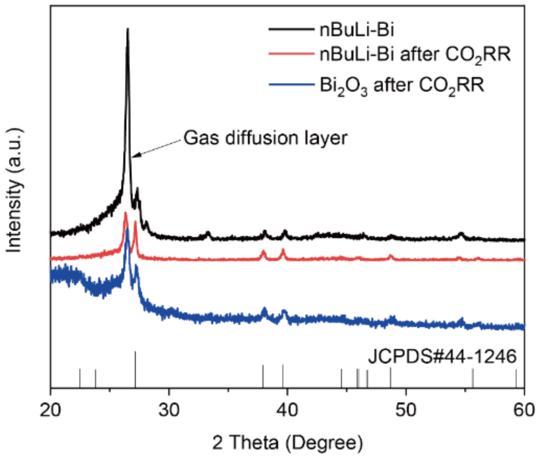

Supplementary Figure 2. **XRD characterizations of Bi_2O_3 and nBuLi-Bi.** XRD pattern of nBuLi-Bi, nBuLi-Bi after CO_2RR, and Bi_2O_3 after CO_2RR. The peaks were consistent with metallic Bi (JCPDS#44-1246).

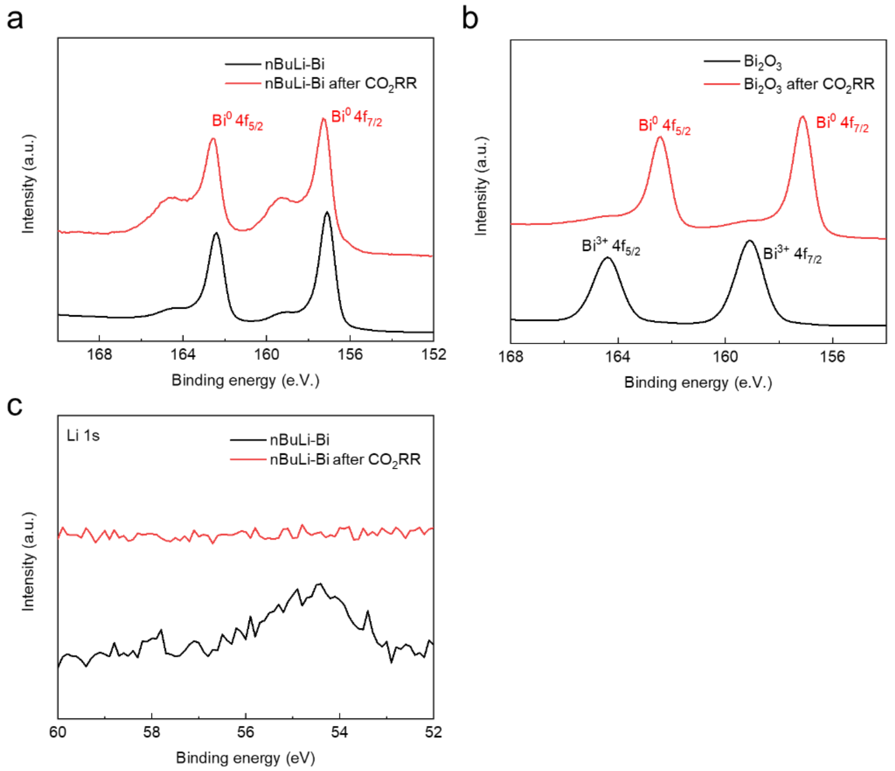

Supplementary Figure 3. **XPS characterizations of Bi_2O_3 and nBuLi-Bi. a**, Ex-situ high resolution Bi 4f spectra for nBuLi-Bi before and after CO_2RR, indicating that Bi_2O_3 have been reduced to metallic Bi by n-butyl lithium. **b**, Ex-situ high resolution Bi 4f spectra for Bi_2O_3 before and after CO_2RR, demonstrating that the oxidized Bi ($4f_{7/2}$ at 159.5 eV) in Bi_2O_3 was almost reduced into metallic Bi ($4f_{7/2}$ at 157 eV) in Bi_2O_3. Note that the residual Bi oxides are due to the oxidation of active Bi when the catalyst was exposed to air during the XPS test. **c**. Ex-situ high-resolution Li 1s spectra for nBuLi-Bi before and after CO_2RR, showing that the nBuLi-Bi surface was free of Li after CO_2RR.

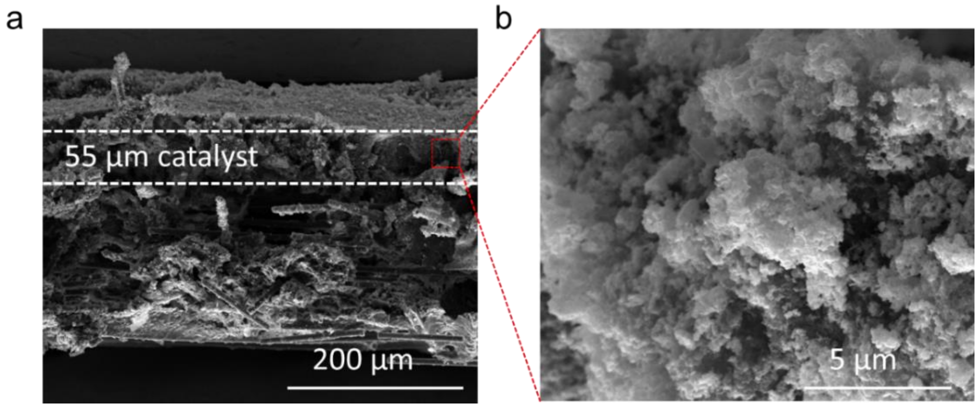

Supplementary Figure 4. SEM characterizations of nBuLi-Bi. SEM images of nBuLi-Bi coated gas diffusion layer. The catalyst layer was *ca.* 55 µm.

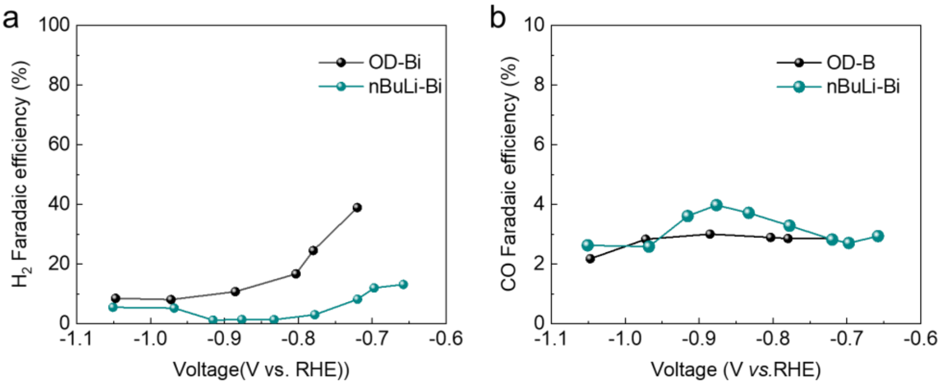

Supplementary Figure 5. **Faradaic efficiencies of byproducts.** Faradaic efficiencies of H_2 and CO in flow cell using 1.0 M $KHCO_3$.

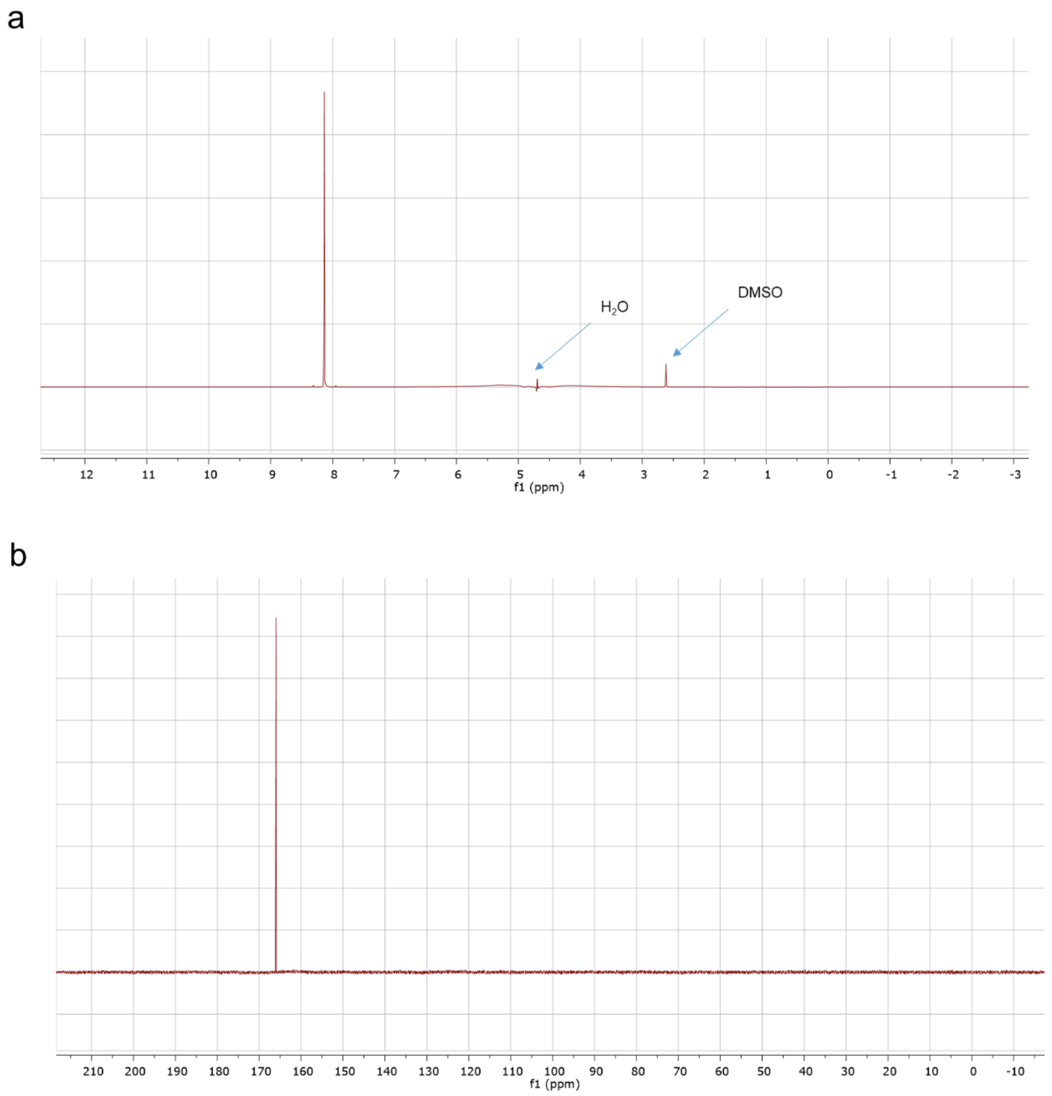

Supplementary Figure 6. NMR spectra of liquid products. Typical (a) ^{1}H and (b) ^{13}C NMR spectra for as-prepared pure formic acid solution, demonstrating that no other liquid products are formed.

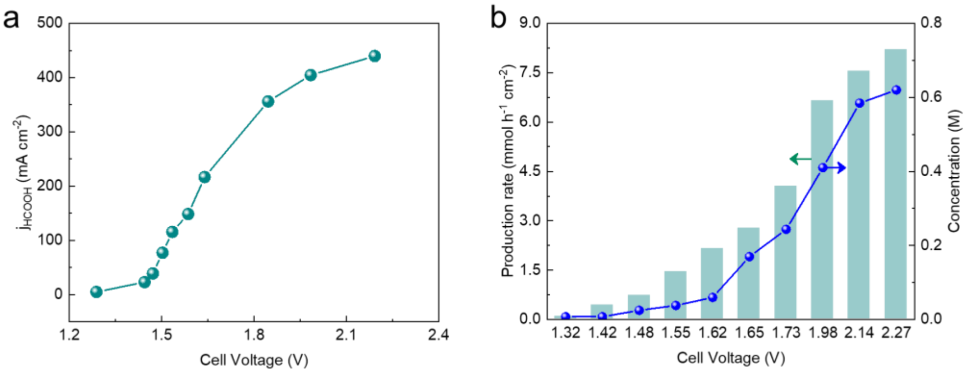

Supplementary Figure 7. Electrochemical performance of nBuLi-Bi using PSE. a, The partial current densities of HCOOH over cell voltages on nBuLi-Bi, where DI water was used to release the produced HCOOH. **b**, The production rate and concentration of pure HCOOH solution on nBuLi-Bi over various cell voltages.

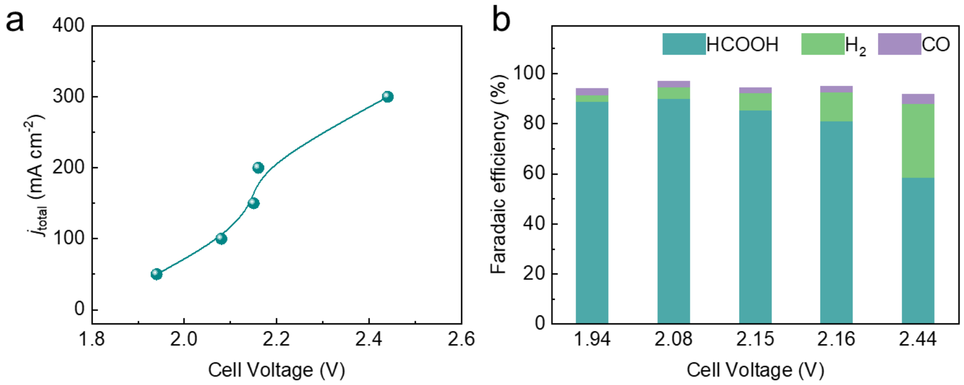

Supplementary Figure 8. CO_2RR performance of OD-Bi using PSE reactor. a, The I-V curve of OD-Bi//PSE//Pt/C cell, where DI water was used to release the produced formic acid molecules. **b**, The corresponding FEs of different products under different cell voltages.

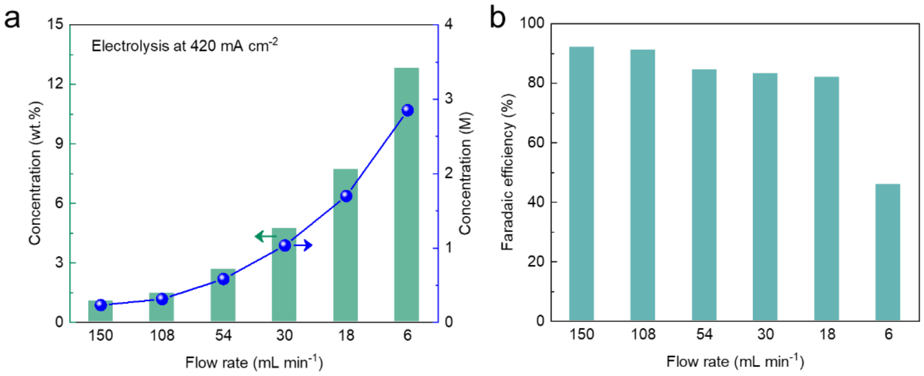

Supplementary Figure 9. DI water flow rate effects. a, The dependence of pure HCOOH solution concentration on the DI water flow rate. **b**, The dependence of HCOOH Faradaic efficiency on the DI water flow rate at an overall current density of 420 mA cm^{-2}.

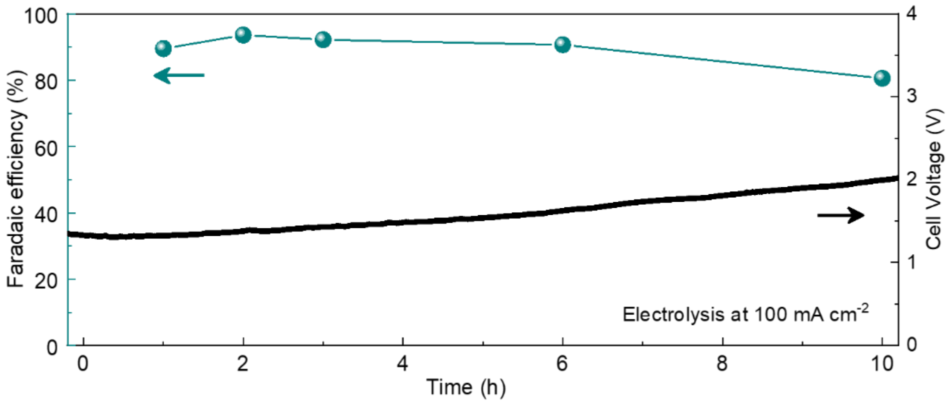

Supplementary Figure 10. **Stability test at high current density**.

Stability test with a fixed current density of 100 mA cm^{-2}.

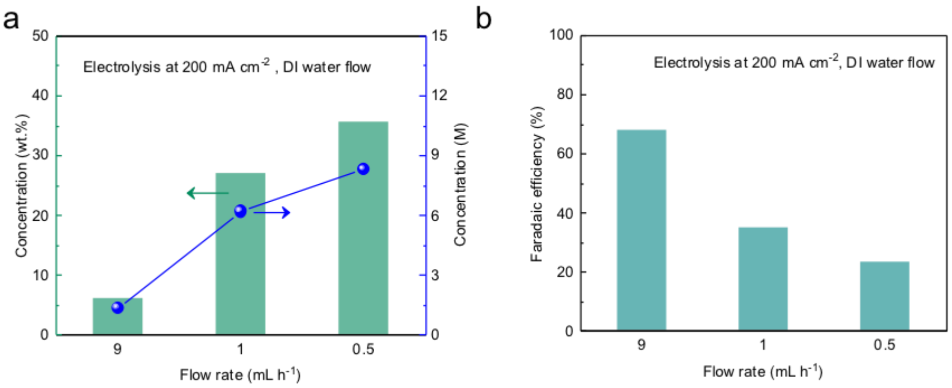

Supplementary Figure 11. Flow rate effects using low DI water flow rates. a, The dependence of pure HCOOH solution concentration on the DI water flow rate at an overall current density of 200 mA cm^{-2}. **b**, The dependence of HCOOH Faradaic efficiency on the DI water flow rate at an overall current density of 200 mA cm^{-2}.

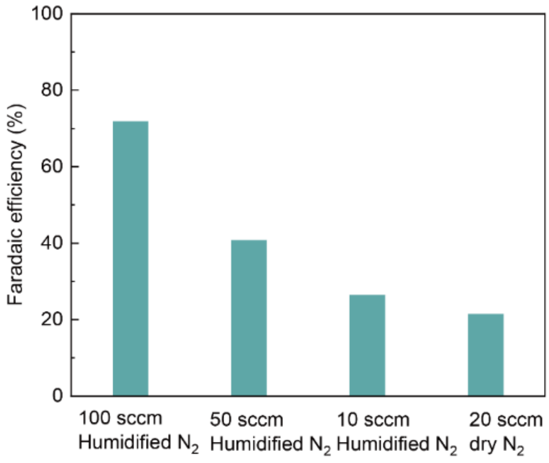

Supplementary Figure 12. Flow rate effects using N_2 gas flow. FEs of pure HCOOH vapour on nBuLi-Bi over various humidified/dry N_2 gas flow rate maintaining an overall current density of 200 mA cm^{-2}.

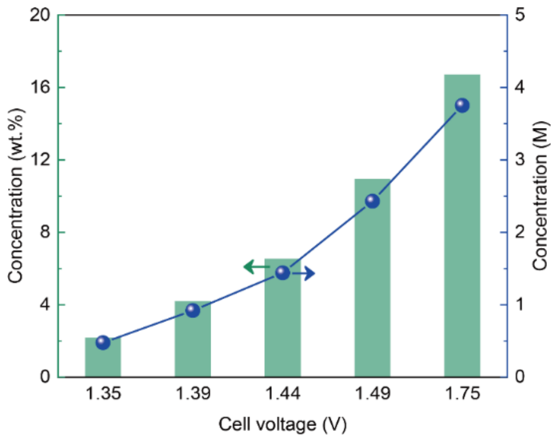

Supplementary Figure 13. Electrochemical performance using gas flow. The concentrations of pure HCOOH vapour over cell voltages on nBuLi-Bi using 100 sccm humidified N_2.

a

b

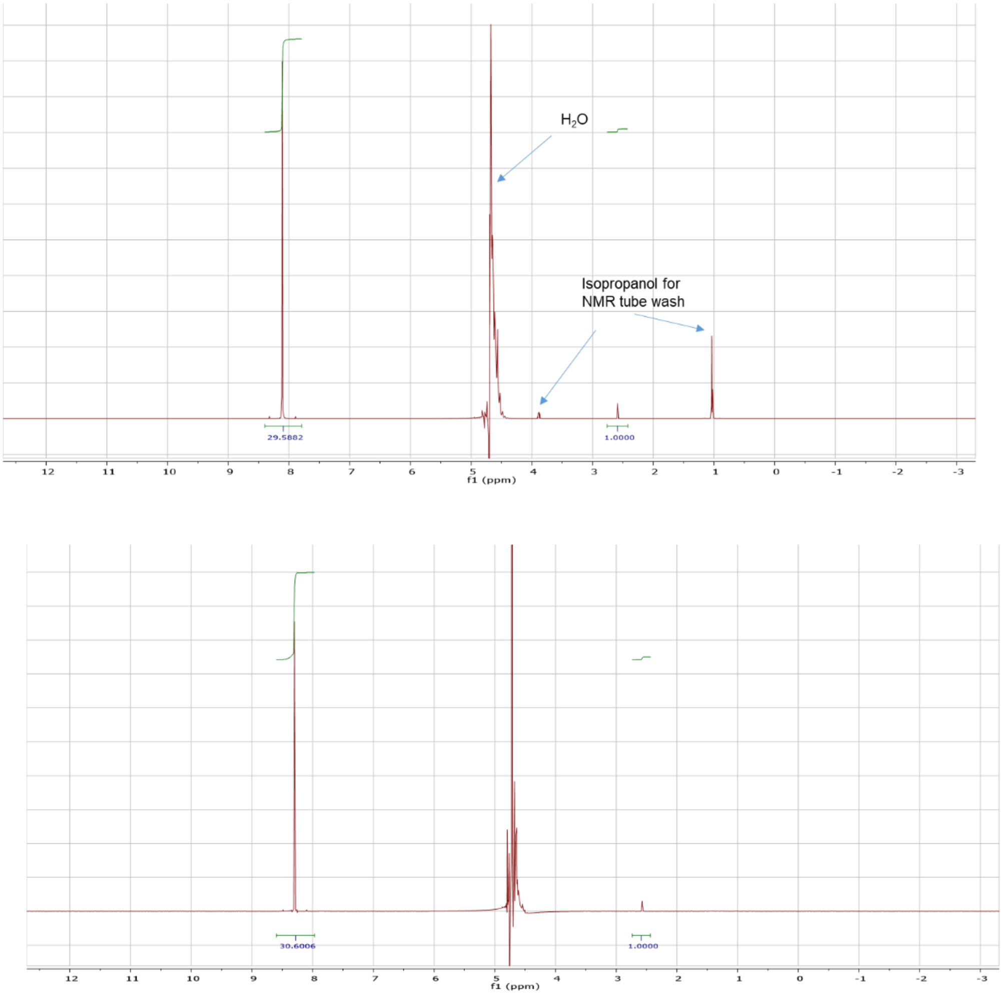

Supplementary Figure 14. NMR spectrum of pure formic acid. The NMR spectrum of commercial formic acid product (**a**) and formic acid product obtained via dry N_2 flow (**b**).

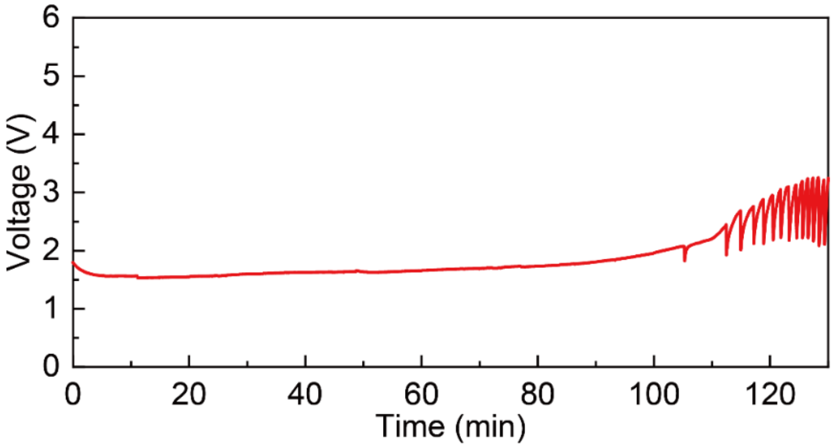

Supplementary Figure 15. The voltage profile using dry N_2 flow. The voltage profile was monitored for the all-solid-state reactor using 20 sccm dry N_2 flow. After ~110 min operating, the voltage begins to fluctuate due to the gradual degradation of membranes and solid electrolytes without wetting conditions.

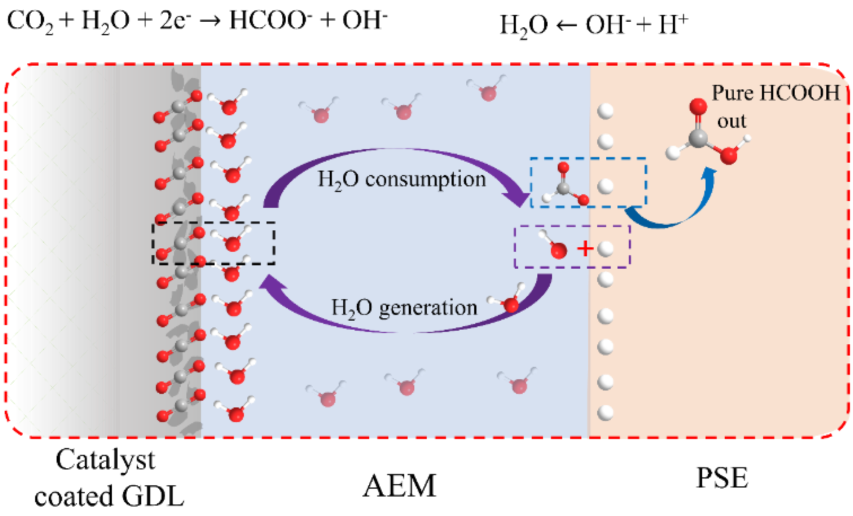

Supplementary Figure 16. Schematic illustration of water consumption and generation in the AEM.

Supplementary Tables

Supplementary Table 1. Comparison of Catalytic Parameters of our methods and Control

Reference	Purity	Max. Concentration (M)	Max. $j_{formate/HCOOH}$ (mA cm^{-2})	Stability	Max. Productivity (mmol h^{-1} cm^{-2})
This Work	Pure	26.7	449	100 h	8.38
1	pure	12.1	172	100 h	3.21
2	Mixture	0.1	206	13 h	3.84
3	Mixture	-	180	60h	3.35
4	Mixture	-	52	40h	0.97
5	Mixture	-	31	50h	0.58
6	Mixture	-	20	10h	0.37

Supplementary Table 2. The concentration of impurities for generated HCOOH using our all-solid-state cell. Note that the reported concentrations are average results acquired from 3 independent tests.

Iron	Potassium	Sodium	Bismuth	Platinum	Sulfur
<0.01 ppm	5.49 ppm	0.20 ppm	<0.01 ppm	<0.01 ppm	0.23 ppm

Supplementary Notes

Supplementary Note 1: Preliminary estimation of the production cost for the electrosynthesis of HCOOH using the 4.75 cm^2 CO_2//SE//H_2 cell. We only calculated the costs of energy and feedstock input; no other costs associated with practical production or infrastructure were included. The market price of formic acid is \$0.74/kg[7].

CO_2//SE//H_2 cell: Operation condition: 1.98 V (3000 mA); Production rate: 7.55 mmol cm^{-2} h^{-1} (1.65 g h^{-1})

$$m_{\mathrm{HCOOH}} = \frac{1\,kWh}{1.98\,V \times 3\,A} \times 1.65\,g\,h^{-1} = \sim 277.8\text{ g} \qquad (1)$$

Thus, we can obtain 0.2778 kg HCOOH using 1 kWh electricity. This 0.2778 kg HCOOH consumes 0.2657 kg CO_2 and 0.012 kg H_2, where the industrial CO2 price is \$0.03/kg, and the price of H_2 is ~ \$3.9/kg [8]. Therefore, the CO_2 cost is 0.8 cents, H_2 cost is 4.7 cents. Assuming the price of electricity is 3 cents/kWh, we can roughly estimate a HCOOH production cost of ca. \$0.31/kg-HCOOH without considering the cost of DI water. The industrial water in Texas is \$1.91 per 1000 gallon (https://www.fbgtx.org/673/IndustrialWater-Rates). Only 1 to 3 cents are needed to deionize one gallon of water (https://blog.uswatersystems.com/2012/08/de-ionization-101/). Thus, the price of DI water is estimated to be ~ 3 cents/gallon or \$0.008/kg, which only adds a marginal cost to the HCOOH production cost of \$0.31/kg.

Supplementary Note 2: Production of pure HCOOH

In our study, electrosynthesis of HCOOH can be decoupled into two half-cell reactions (Supplementary Eq. 2 and 3), followed by the ionic recombination process (Supplementary Eq. 4):

CO_2 reduction reaction: $CO_2 + H_2O + 2e^- \rightarrow HCOO^- + OH^-$ (2)

H_2 oxidation reaction: $H_2 \rightarrow 2H^+ + 2e^-$ (3)

Recombination in PSE: $HCOO^- + OH^- + 2H^+ \rightarrow HCOOH + H_2O$ (4)

Total reaction: $CO_2 + H_2 \rightarrow HCOOH$ (5)

So, in principle, there is no need for extra water. As we have mentioned in the experimental section that dry CO_2, N_2, and H_2 were used for pure HCOOH vapour (almost 100%) production to avoid the introduction of any water vapor. In this system, CO_2RR is performed at the cathode, making one $HCOO^-$ (formate) and one OH^-, and simultaneously consuming one water (Supplementary Eq. 2). Since only dry CO_2 is provided, the surface water of the AEM membrane will involve in the electrochemical CO_2 reduction process to initiate the CO_2-to-formate conversion[9]. Next, the generated $HCOO^-$ and OH^- will transport through the AEM into the porous PSE layer to combine with the two H^+ to produce one HCOOH (formic acid) and one H_2O. This recommendation process will occur at the AEM/PSE interface since a solid proton conductor was used in our case[1]. Then, the recombined H_2O will be immediately absorbed by the surface imidazole groups of the AEM membrane[10]. Thus, no net water was generated in the entire electrochemical CO_2 reduction cycle. What is more, while trace amount of water vapor will be introduced into the system from environmental moisture, the AEM, CEM and porous PSE is superhydrophilic, which can absorb the water molecules. Moreover, compared with water, formic acid is more volatile, which makes it easier to be released by the nitrogen flow. All the features, take together, make the production of ultrapure HCOOH vapor possible.

Supplementary References

1 Xia, C. *et al.* Continuous production of pure liquid fuel solutions via electrocatalytic CO_2 reduction using solid-electrolyte devices. *Nature Energy* **4**, 776-785 (2019).

2 Gong, Q. *et al.* Structural defects on converted bismuth oxide nanotubes enable highly active electrocatalysis of carbon dioxide reduction. *Nat. Commun.* **10**, 2807 (2019).

3 García de Arquer, F. P. *et al.* 2D metal oxyhalide-derived catalysts for efficient CO2 electroreduction. *Advanced Materials* **30**, 1802858 (2018).

4 Zheng, X. *et al.* Sulfur-modulated tin sites enable highly selective electrochemical reduction of CO_2 to formate. *Joule* **1**, 794-805 (2017).

5 Zheng, X. *et al.* Theory-guided Sn/Cu alloying for efficient CO_2 electroreduction at low overpotentials. *Nature Catalysis* **2**, 55-61 (2019).

6 Han, N. *et al.* Ultrathin bismuth nanosheets from in situ topotactic transformation for selective electrocatalytic CO_2 reduction to formate. *Nat. Commun.* **9**, 1320 (2018).

7 Jouny, M., Luc, W. & Jiao, F. General techno-economic analysis of CO_2 electrolysis systems. *Industrial & Engineering Chemistry Research* **57**, 2165-2177 (2018).

8 De Luna, P. *et al.* What would it take for renewably powered electrosynthesis to displace petrochemical processes? *Science* **364**, eaav3506 (2019).

9 Zheng, Y. *et al.* Water uptake study of anion exchange membranes. *Macromolecules* **51**, 3264-3278 (2018).

10 Amini, S., Hadipour, N. & Elmi, F. A study of hydrogen bond of imidazole and its 4-nitro derivative by ab initio and DFT calculated NQR parameters. *Chemical physics letters* **391**, 95-100 (2004).
